# Different motif requirements for the localization zipcode element of β-actin mRNA binding by HuD and ZBP1

**DOI:** 10.1093/nar/gkv699

**Published:** 2015-07-07

**Authors:** Hak Hee Kim, Seung Joon Lee, Amy S. Gardiner, Nora I. Perrone-Bizzozero, Soonmoon Yoo

**Affiliations:** 1 Nemours Biomedical Research, Alfred I. duPont Hosp. for Children, Wilmington, DE 19803, USA; 2 Department of Biological Sciences, University of South Carolina, Columbia, SC 29208, USA; 3 Department of Neuroscience, University of New Mexico School of Medicine, Albuquerque, NM 87131, USA

## Abstract

Interactions of RNA-binding proteins (RBPs) with their target transcripts are essential for regulating gene expression at the posttranscriptional level including mRNA export/localization, stability, and translation. ZBP1 and HuD are RBPs that play pivotal roles in mRNA transport and local translational control in neuronal processes. While HuD possesses three RNA recognition motifs (RRMs), ZBP1 contains two RRMs and four K homology (KH) domains that either increase target specificity or provide a multi-target binding capability. Here we used isolated *cis*-element sequences of the target mRNA to examine directly protein-RNA interactions in cell-free systems. We found that both ZBP1 and HuD bind the zipcode element in rat β-actin mRNA's 3′ UTR. Differences between HuD and ZBP1 were observed in their binding preference to the element. HuD showed a binding preference for U-rich sequence. In contrast, ZBP1 binding to the zipcode RNA depended more on the structural level, as it required the proper spatial organization of a stem-loop that is mainly determined by the U-rich element juxtaposed to the 3′ end of a 5′-ACACCC-3′ motif. On the basis of this work, we propose that ZBP1 and HuD bind to overlapping sites in the β-actin zipcode, but they recognize different features of this target sequence.

## INTRODUCTION

The subcellular localization of RNAs has emerged as an important molecular mechanism that is evolutionarily conserved for restricting certain transcripts and proteins to a specific subcellular locale. Current evidence suggests that axonal mRNA transport and local protein synthesis are required for new growth cone formation, retrograde signaling, axon guidance, and elongation (1,2). Elucidating axonal mRNA transport mechanisms would enable researchers to discover ways to stimulate or reintroduce adult CNS neurons with the capacity to regenerate.

mRNA transport into axons is directly mediated by localization *cis*-elements mostly present within the untranslated regions (UTRs) of the mRNAs, which are specifically recognized by *trans*-acting RNA-binding proteins (RBPs), and assembled into large ribonucleoprotein (RNP) complexes (3). For example, β-actin mRNA transport is accomplished by targeting mechanisms involving the zipcode *cis*-element within the 3′ UTR and its corresponding zipcode binding protein 1 (ZBP1) (4–6). The AU-rich regulatory elements (AREs) within the 3′ UTR of Tau and GAP-43 mRNAs provide binding sites for the ELAV-like/Hu family protein HuD to be targeted into distal axons (7–9). *Trans*-acting RBPs must interact with a specific target RNA molecule for its localization by recognizing specific sequences or structural features of the RNA, or both. The perturbation in the specificity of RBP-RNA interactions can be directly implicated in a number of diseases that result from perturbed RNA localization. Therefore, understanding the binding mechanisms of RBP-RNA interactions and their specificities has become a topic of great interest.

Over the last decade, several computational approaches have been developed to predict RBP-RNA interactions (10,11). However, the specific binding mechanism is not always supported by experimental investigations, possibly because of different binding preferences. Some RBPs rely solely on well-defined sequences of their target RNA for recognizing and stabilizing the RBP-RNA interaction to play many essential cellular reactions, whereas others capture the structural features of their target RNA that is induced by RNA sequences. Currently, the detection and verification of specific binding preference is only possible by carrying out *in vitro* and *in vivo* experiments. However, few studies have attempted to identify and determine the precise binding preferences of different RBPs for the same target RNA.

Recent advances in cross-linking immunoprecipitation (CLIP)-based technologies have enabled the study of RBP–RNA interactions on a genomic scale at high resolution (12–15). Although these methods allow researchers to directly sequence the RBP-bound sites of the RNAs, the identification of RNA sequences alone cannot explain the specificity of RBP–RNA interactions. In addition, such relatively short sequential motifs that can be present in many RNAs lead to high false positives, complicating the interpretation of the data in determining molecular specificity of RBP–RNA interactions. Meanwhile, conventional RNA-protein pull-down studies on RBP–RNA interactions have typically identified the binding partner RNAs of a single RBP. For example, ZBP1 is known to have many different mRNAs as binding partner RNAs, including β-actin, tau, c-myc and Igf2 (16,17). Another RBP, HuD, recognizes and binds to AU- or pyrimidine-rich RNA sequences of many RNA targets, such as GAP-43, tau, cpg 15 (also called neuritin) and c-myc (18). Although the set of target RNAs can often be determined experimentally by methodologies above, identifying bound RBPs is much more challenging, as a single RNA can bind to more than one RBP directly or indirectly. Therefore, systematic experimental investigations on binding preferences of different RBPs for the same RNA target will be needed to search for relevant sequences or structural motifs, or both, within the RNA sequences.

Recent findings indicate that both ZBP1 and HuD can associate with β-actin mRNA and direct its transport, stability, and translation (4,19–21). Although their binding modes and preferences remain largely elusive, it has been suggested that RNA sequences in the zipcode element are directly involved in the formation of RNP complexes. Whether ZBP1 and HuD share the same sequential features in the zipcode element has not yet been fully investigated. Here, we characterize and analyse the binding preference of ZBP1 and HuD toward the zipcode element present within the 3′ UTR of β-actin mRNA. We demonstrate that HuD recognizes and binds the zipcode RNA with a simple preference toward the primary sequence, a conserved U-rich motif, but ZBP1 is capable of recognizing the structural features of the RNA *cis*-element both specifically and directly. On the basis of our results, we demonstrate a direct comparison for substrate recognition and binding by the HuD and ZBP1 proteins in an apparently mutually exclusive manner.

## MATERIALS AND METHODS

### Animal care

All animal protocols were approved by the Institutional Animal Care and Use Committee, and the experiments were conducted under the IACUC at Alfred I. duPont Hospital for Children.

### Cell culture and transfections

L2–6 DRGs were dissociated with 50 U collagenase type XI (Sigma) for 25 min at 37°C and then triturated with a fire-polished Pasteur pipette. Dissociated DRG neurons were suspended in ‘nucleofector solution’ (Lonza), and 5–7 μg plasmid was electroporated using an AMAXA 4D-Nucleofector system (Lonza). Complete culture medium in DMEM:F12 (Life Technologies), 1x N1 supplement (Sigma), 5% horse serum, 5% fetal bovine serum (Hyclone), and 10 μM cytosine arabinoside (Sigma) was directly added into the transfection cuvette, and DRG neurons were plated on coverslips coated with poly-l-lysine (Sigma) and laminin (Millipore) at 37°C, 5% CO_2_.

### Protein purification

Coding region of rat HuD protein was cloned into pGEX-2T GST construct (GE Healthcare Life Sciences), and rat ZBP1 protein sequence was cloned into the same construct and GST was replaced with 6X His for further purification. An overnight culture of *Escherichia coli* BL 21 (NEB) transformed with pGEX-GST-HuD plasmid was diluted 1:50 in LB medium. At an OD_600_ of 0.4, the culture was induced with isoproryl-β-_D_-thiogalactopyranoside (IPTG, 0.1 mM) (Life Technologies) at 37°C. Four hours later, the cells were spun down and resuspended with purification buffer (20 mM Tris pH 8.0, 500 mM sodium chloride, 100 mM EDTA). The cells were lysed by the addition of lysozyme (0.2 mg/ml) and sonication. The lysate was incubated at 4°C for 15 min with 1% Triton X-100 (v/v) and 1% sarkosyl (w/v) for solubilisation. It was then centrifuged 16 000 *g* for 15 min, and the resulting supernatant was collected and passed through 45 μm filter membrane. GST-Sepharose 4B beads (GE Healthcare Life Sciences) were used to purify GST-HuD protein. Purified GST protein was eluted with 20 mM glutathione in 50 mM Tris–HCl pH 8.0, desalted using centrifugal filter unit.

His-ZBP1 was purified as described above with slight modification. In brief, *E. coli* BL 21 transformed with pGEX-His-ZBP1 plasmid was induced with 0.1 mM of IPTG at 27°C for 6 h. After spinning down, cell pellet was re-suspended with buffer (50 mM sodium phosphate, 500 mM sodium chloride, 10 mM imidazole). Ni-NTA beads (Life Technologies) were used for purification. Purified His-ZBP1 protein was eluted with 250 mM imidazole.

Bradford and BCA assays (Bio-Rad) were used for quantification of GST-HuD and His-ZBP1 proteins, respectively. Concentration of the purified proteins was confirmed by comparison with bovine serum albumin (BSA) standards in acrylamide gel stained with Coomassie brilliant blue. Purity after purification was evaluated by Coomassie brilliant blue staining, and the sample with higher than 95% purity was used for binding assays.

### RNA binding assays

Purified GST-HuD and His-ZBP1 proteins were probed with Dynabead Protein G (Life Technologies) conjugated with anti-GST and anti-His antibody (Life technologies). Beads conjugated with mouse IgG were used to see the background binding. Beads were washed with binding buffer (Promega) and incubated with 1 μg of total RNA isolated from rat dorsal root ganglion (DRG) in binding buffer (10 mM HEPES, 3 mM MgCl_2_, 40 mM KCl, 1 mM DTT, 400 U of RNaseOUT™ and 5% glycerol) at 4°C for 30 min. After washing, beads were incubated in TRIzol (Life Technologies) and extracted by phenol–chloroform to isolate RNA. RNA was precipitated with ethanol using a glycogen carrier.

### RNA probe preparation and RNA electrophoretic mobility shift assays (REMSA)

The pcDNA3 plasmid (Life Technologies) containing the full-length 3′ UTR of rat β-actin mRNA was linearized with XhoI restriction endonuclease and transcribed *in vitro* from the SP6 promoter using Riboprobe^®^ In Vitro Transcription Systems (Promega) as described in the technical manual. The transcribed RNAs were biotinylated on their 3′ end using RNA 3′ End Biotinylation Kit (Thermo Scientific Pierce). Ribogreen assay (Life Technologies) was used to quantify concentration of RNA, and dot blot was performed to confirm the biotinylation efficiency. The 54 nucleotides zipcode motifs from rat and mouse mRNA and zipcode mutants were customized from IDT (Integrated DNA Technologies). For binding assay, RNAs were biotinylated at both ends. In some experiments, an excess of cold zipcode RNA was used to confirm the specificity of the assays. A reaction mixture (20 μl) containing 20 fmol of RNA and 3 pmol of purified protein was incubated in a binding buffer (50 mM Tris–HCl, 150 mM NaCl, 0.25 mg/ml tRNA, 0.25 mg/ml bovine serum albumin, 0.2 mM dithiothreitol, and 5 mM MgCl_2_, pH 7.0) at room temperature for 20 min. After incubation, the binding reaction was resolved on a native 6% polyacrylamide gel in 0.5× TBE and transferred to a nylon membrane. Band shifts were detected using the Chemiluminescent Nucleic Acid Detection Module (Pierce).

The apparent dissociation constant (*K*_d, apparent_) between the wild-type and mutant RNA for ZBP1 was obtained using three independent REMSA experiments. The fraction of bound RNA was plotted graphically versus protein concentration, and the binding capability of ZBP1 for the wild-type RNA and mutant was compared. Nonlinear regression fits of the data revealed an apparent dissociation constant equal to 28.1 nM for wild-type RNA, statistically different from the *K*_d, apparent_ = 315.4 nM for the mutant RNA (*P* < 0.0001).

Secondary structure of RNA was predicted using web-based software, mFold (http://mfold.rna.albany.edu/) and confirmed again with Vienna Fold (http://rna.tbi.univie.ac.at/cgi-bin/RNAfold.cgi). In case RNA shows more than one prediction, the structure with the lowest Δ*G* was considered as main structure.

### RNA structural probing with RNase III

ShortCut^®^ RNase III (NEB) was used for RNA cleavage assays. The enzyme cleaves dsRNA into short dsRNA fragments (18–25 bp) in a manganese-containing reaction buffer. Cleavage reactions were performed in 20 μl volumes containing 10 fmol of biotin-labelled wild-type or mutant zipcode RNAs. 0.4 or 4 units of RNase III were added to the reaction and incubated at 37°C for 20 min. EDTA was added to stop the reaction and immediately loaded onto 6% denature acryl amide gel (7 M urea, 1× TBE) after mixing with 2× denaturing loading buffer (95% formamide, 18 mM EDTA, 0.025% SDS, 0.1% xylene cyanol and 0.1% bromophenol blue). After running, each gel was transferred onto positively charged nylon membrane and developed as described above in REMSA method.

### *In vitro* mRNA stability assay

S100 extracts were prepared from cortical tissue of *HuD^−/−^* mouse (HuD KO) according to Bird *et al*. (22). To examine the biological role of HuD protein on β-actin mRNA, 30 μg of the HuD KO S100 extract was incubated with 300 ng of total RNA from wild-type mouse cortex and incubated at 37°C for various time-points. In a separate series, 75 ng of purified HuD protein was added to each decay reaction. RNA was isolated from the decay reactions with or without exogenous HuD protein using phenol-chloroform extraction followed by ethanol precipitation. After *DNaseI*treatment for 10 min at 37°C, 200 ng of RNA was used to generate cDNA using *Superscript II* reverse transcriptase (Invitrogen) and processed further for quantitative PCR (qPCR) using an Applied Biosystems 7300 Real-Time PCR System. β-Actin mRNA signals were normalized to GAPDH mRNA using the comparative threshold (*C*_t_) methods and expressed as fold change relative to *t* = 0 min from the 2^−(ΔΔ*C*t)^ calculations. The primers used for were: β-actin sense, 5′-GACGGCCAGGTCATCACTAT-3′ and antisense, 5′-CTTCTGCATCCTGTCAGCAA-3′; GAPDH sense, 5′-TGTGATGGGTGTGAACCACGAGAA-3′ and antisense, 5′-GAGCCCTTCCACAATGCCAAAGTT-3′.

### Fluorescent *in situ* hybridization (FISH)

The wild-type and mutants of zipcode sequence were incorporated into the *pcDNA3* (Invitrogen). The plasmid was linearized with restriction endonuclease and transcribed *in vitro* using Riboprobe^®^ In Vitro Transcription Systems (Promega) to generate cRNA probe for *in situ* hybridization as described in the technical manual. Antisense probe for eGFP reporter was transcribed from SP6 promoter and sense probe was transcribed from T7 promoter. Probes were chemically labelled with digoxigenin succinamide ester (Roche) during *in vitro* transcription. cRNAs were then alkaline hydrolysed to 100–150 nt length. All probes were stored at −80ºC until used.

FISH for DRG cultures was similar to previously described methods (21). All steps were carried out at room temperature unless otherwise indicated. Coverslips were rinsed in phosphate buffered saline (PBS), fixed in buffered 2% paraformaldehyde for 20 min, and then permeabilized in 0.3% Triton X-100 for 5 min. The coverslips were then rinsed in PBS, and hybridization was performed at 55ºC for 1 h in 5× SSC, 50% formamide, 20% dextran sulphate, 25 μg/ml salmon sperm DNA, 25 μg/ml *E. coli* tRNA, and 0.5× Denhardt's solution containing 5 ng cRNA per coverslip. Coverslips were then washed twice for 20 min in 50% formamide/2× SSC at 37ºC, followed by three washes in 1× SSC for 10 min each on a rotary shaker.

For subsequent IF, samples were blocked in 50 mM Tris, 150 mM NaCl, 2% BSA, and 2% fetal bovine serum (‘IF buffer’) for 1 h. After blocking, samples were incubated for 1 h in the following primary antibodies in IF buffer: chicken anti-neurofilament (NF) H (1:1000; Chemicon), chicken anti-peripherin (1:1500; Millipore), and sheep anti-digoxigenin (1:1000; Roche). Samples were washed in TBS buffer three times and then incubated with Alexa633-conjugated anti-chicken IgG antibody (1:2000; Life Technologies) and Alexa488-conjugated anti-sheep IgG antibody (1:2000; Life Technologies) in IF buffer for 1 h. Coverslips were mounted using PVA-DABCO (Sigma) anti-fading mounting medium and visualized with Leica DMRXA2 epifluorescent microscope. All images were matched for acquisition parameters.

### Statistical analyses

Graphpad Prism 6 software package (GraphPad) was used for statistical analyses. Non-linear regression analyses were used for the competitive binding experiments, the apparent dissociation constant between the wild-type, mutant, and chicken RNAs for ZBP1, and β-actin decay experiments.

## RESULTS

### Both HuD and ZBP1 bind β-actin mRNA with a binding specificity in the mRNA localization signal within the 3′ UTR

Our recent studies using quantitative real-time PCR (qRT-PCR) showed that β-actin and GAP-43 RNAs were detected both in the HuD immunoprecipitate and ZBP1 immunoprecipitate with relatively different levels of enrichment, suggesting a different affinity of these proteins for different mRNAs (21). However, precipitation of RNAs present in whole cell lysate by antibodies does not eliminate the apparent possibility that other proteins present in the lysate could form messenger ribonucleoprotein (mRNP) complex with the immunoprecipitated proteins *via* direct or indirect interaction. Furthermore, there have been relatively few studies for a direct comparison of the binding preference of different proteins to the same RNA target. We hypothesized that differential binding preference of different RBPs toward the same target RNA would provide an additional mechanism to increase specificity of mRNA localization into distal processes of neurons. To ascertain that the precipitation of β-actin mRNA with ZBP1 or HuD protein that we have previously shown is in fact dependent on direct protein–RNA interaction, rather than through indirect protein-protein interactions, both full ZBP1 and HuD were expressed and purified (Supplementary Figure S1). Briefly, whole cell lysate of *E. coli* BL15 strain expressing either GST-tagged HuD (HuD^GST^) or His-tagged ZBP1 (ZBP1^His^) was incubated with Glutathione-Sepharose beads or Ni-nitrilotriacetic acid (Ni-NTA) beads, respectively, followed by removal of nonspecific proteins with extensive washing. After eluting, the proteins were concentrated, quantified, and analyzed by SDS-PAGE and western blotting (Supplementary Figures S1A and S1B).

**Figure 1.**
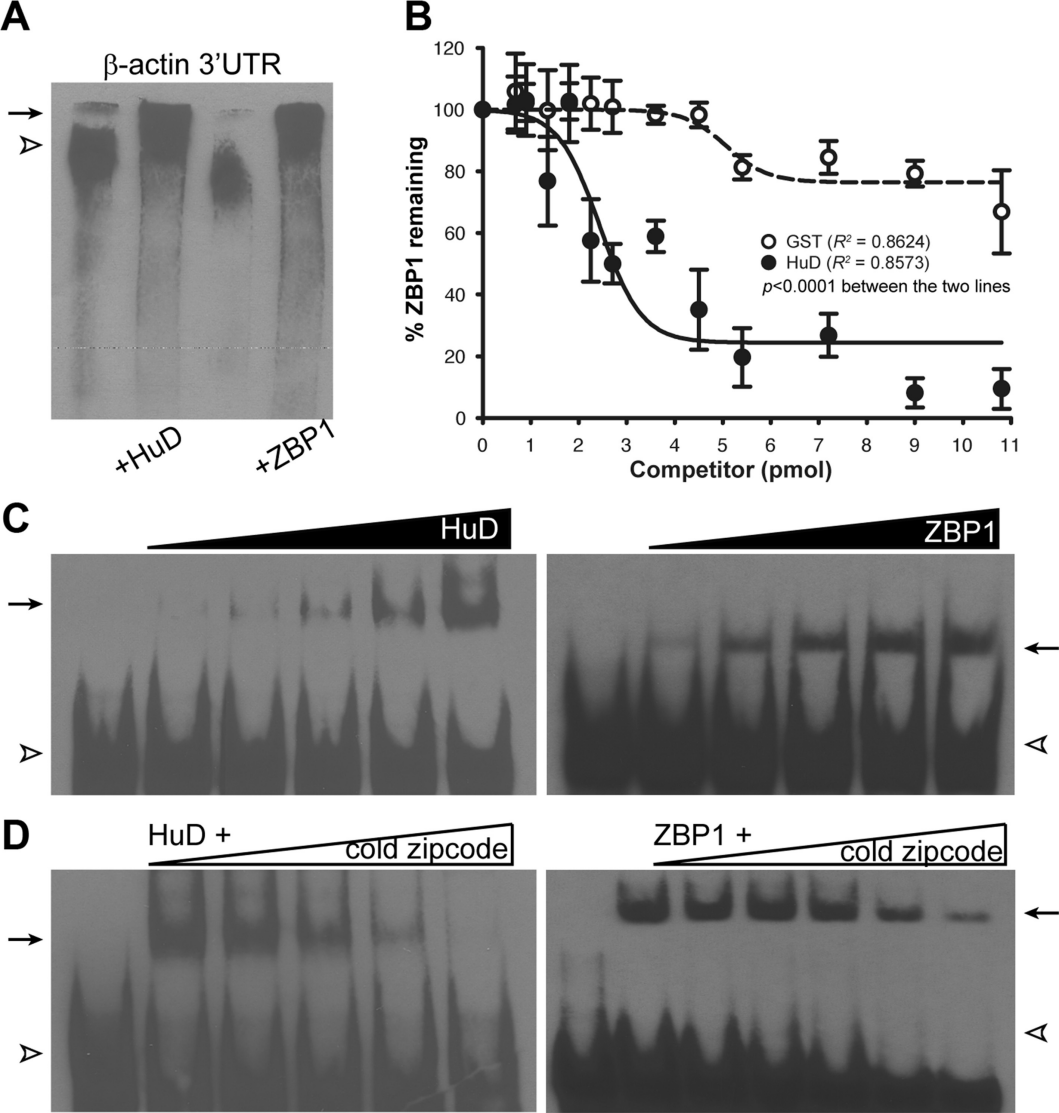
Binding of the rat zipcode RNA to purified HuD and ZBP1 proteins examined by electrophoretic mobility shift assay (EMSA). (**A**) Representative RNA-EMSA (REMSA) gel showed purified HuD and ZBP1 binding to biotin-labelled full-length 3′ UTR of rat β-actin mRNA. Biotin-labelled free RNA (open arrowhead) and RNA-protein complexes (arrow) were indicated. (**B**) Quantification of competitive binding assay of HuD and ZBP1 revealed that increasing amounts of HuD protein in the reaction containing the ZBP1-zipcode RNA complex displaced bound ZBP1 from the complex. Error bars represented standard error of the mean (*n* > 3). The measured values were fitted to a non-linear regression curves, resulting in a significant difference in the slopes of regression lines between HuD and GST (*P* < 0.0001). (**C**) and (**D**) Similar to (A) but the full-length 3′ UTR of rat β-actin mRNA was replaced with biotin-labelled synthetic zipcode RNA. The triangles on the top of the images represented a serial dilution of purified proteins (C) and unlabeled RNA target (D).

ZBP1 is one of the best-studied *trans*-acting factors that specifically recognizes a 54-nt sequence in the 3′ UTR of chicken β-actin mRNA, called the zipcode that is required and sufficient for its subcellular localization and translational regulation (4–6). Although several studies have suggested a role of HuD for β-actin mRNA localization and regulation of axonal actin dynamics (19–21), we know surprisingly little about how HuD recognizes and binds to β-actin mRNA. Therefore, we first focused on the interaction of HuD and the full-length 3′ UTR of β-actin mRNA and compared the results with those with ZBP1. To test whether HuD directly binds β-actin mRNA's 3′ UTR, the full-length 3′ UTR was transcribed *in vitro* then biotinylated. The RNA electrophoretic mobility shift assay (REMSA) was conducted to resolve the biotin-labeled 3′ UTR in complex with purified HuD and compared with ZBP1-β-actin 3′ UTR complex (Figure 1). Initial REMSA experiments using the *in vitro* transcribed full-length 3′ UTR of β-actin mRNA showed a direct binding of purified HuD and ZBP1 proteins with roughly similar affinity (Figure 1A). However, this did not distinguish binding sites on the RNA between ZBP1 and HuD. Further, we were not able to resolve HuD-3′ UTR complex from ZBP1–3′ UTR complex on gels despite altering acrylamide gel concentration and electrophoresis parameters. The information available from such electrophoretic separation of the complexes was mainly limited by the fact that the *in vitro* transcribed full-length 3′ UTR was relatively large (∼0.8 kb).

To further map out binding sites of HuD on the RNA sequences and distinguish HuD–RNA complex from ZBP1-RNA complex on gels, the β-actin mRNA's 3′ UTR was analyzed using bioinformatics algorithms to search for putative HuD-binding sites. Previous studies have shown that the biological functions of HuD result from its ability to recognize and bind to ARE motifs in specific target mRNAs (20–21,23–25). Therefore, we initially focused on bioinformatics analyses for well-known HuD-binding motifs (e.g. AREs) in the 3′ UTR of β-actin mRNA using RegRNA 2.0, a web-based server for identifying regulatory RNA motifs and elements (26). The 3′ UTR contained no known ARE motifs. ARE motifs usually consist of A- and U-rich stretches, as their name indicates. However, these *cis*-acting elements are in fact quite varied in sequence (20,27–28). In addition, several studies demonstrated that all ELAV-like/Hu protein members, including HuR and HuD, preferentially bind U-rich sequences, mostly located in the 3′ UTRs of their target mRNAs (15,29–31). These data imply that the typical ARE motifs may not be necessary for HuD binding to β-actin mRNA's 3′ UTR. Interestingly, our analyses revealed that 3′ UTRs of β-actin mRNAs from mammals examined contained a conserved U-rich sequence within the zipcode sequence (Supplementary Figure S2, shaded in blue), and that deletion of the U-rich motif completely abolished the ability of HuD and ZBP1 binding to the RNA (Figure 4E, ΔU-rich). This raises the possibility that the zipcode element within the β-actin mRNA's 3′ UTR offers binding sites for both ZBP1 and HuD.

To directly test if HuD binds the zipcode element, we analyzed binding of HuD to the RNA using the purified proteins and synthetic wild-type zipcode RNA of the rat β-actin 3′ UTR. As shown in Figure 1C, purified HuD bound the wild-type zipcode with relatively high affinity, comparable with that of ZBP1. These results indicated that the zipcode element present within the β-actin mRNA's 3′ UTR is sufficient for HuD binding to the RNA, as for ZBP1 binding. The specificity of the purified HuD- and ZBP1-zipcode RNA complexes was confirmed by competition assays with a 200-fold molar excess of unlabeled zipcode RNA. As shown in Figure 1D, increasing amount of competitor cold zipcode RNA up to 200-fold of labeled RNA caused a complete inhibition of the shifted complex in the gel. In contrast, a larger than 200 molar excess of unlabeled γ-actin 3′ UTR, which is known to carry no localization *cis*-element, did not have an effect on the complex formation of the zipcode and purified proteins (Supplementary Figure S3).

**Figure 2.**
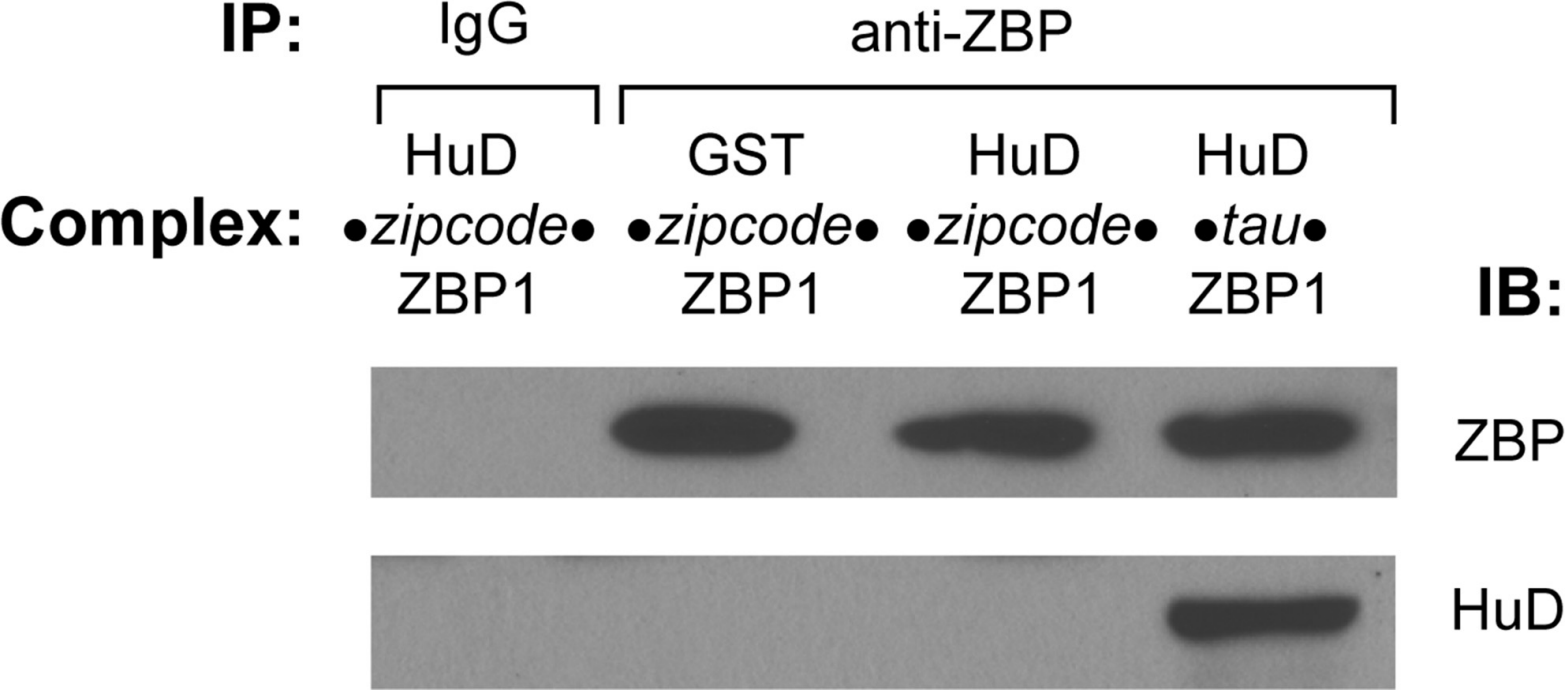
Competitive binding of HuD and ZBP1 to the zipcode RNA. Equimolar amounts of HuD or GST protein were incubated with ZBP1 protein in the presence of the synthetic zipcode RNA for 30 min at room temperature to allow the formation of RBP–RNA complexes. To test the co-immunoprecipitation system (co-IP), tau RNA was included as a positive control. Then the complexes were further incubated with IgG- or anti-ZBP1 antibody-coupled Dynabead Protein G for 2 h to form IP complexes. After extensive washing, the protein-RNA complexes bound to the beads were loaded onto a 10% polyacrylamide gel and analysed by immunoblotting. Although ZBP1 protein was present in the complexes, HuD did not co-immunoprecipitate with ZBP1 *via* the zipcode RNA, compared to co-IP of HuD with ZBP1 via tau RNA. These results suggested that ZBP1 and HuD binding to the RNA is mutually exclusive.

Given that both HuD and ZBP1 bind to the same zipcode element in the β-actin 3′ UTR, we next examined if HuD competes with ZBP1 for the binding to the RNA. For these studies, biotin-labelled synthetic zipcode RNA was incubated with 4 pmol of ZBP1 protein and then additional incubation with increasing amounts of either GST (open circle) or HuD (closed circle) (Figure 1B). The amounts of ZBP1 remained to be bound to the RNA at the end of reaction was subsequently measured by densitometry. As shown in Figure 1B, GST protein did not significantly interfere with ZBP1 binding to the zipcode RNA, but increasing amounts of HuD significantly displaced bound ZBP1 from the zipcode RNA. Based on the data, the calculated inhibitory concentration 50% (IC50) for HuD displacement of ZBP1 was 171.3 nM. To evaluate the possibility that both ZBP1 and HuD could interact with the zipcode RNA simultaneously, we carried out a co-immunoprecipitation (co-IP) assay followed by western blot analysis to visualize the proteins bound to the beads with anti-HuD and anti-ZBP1 antibodies (Figure 2). To this end, equal molar amounts of either HuD or GST (control) protein were first incubated with ZBP1 protein in the presence of the synthetic zipcode RNA simultaneously to allow the formation of RNP complexes. To verify the association of purified HuD and ZBP1 proteins with a specific target RNA molecule, we used the *G fragment* from the tau mRNA's 3′ UTR as a positive control. The 600-nt *G fragment* has been shown to contain direct binding sites for both ZBP1 and HuD (7,32–33). Then, the protein-RNA complexes formed were further incubated with IgG- or anti-ZBP1 antibody-coupled Dynabead Protein G to pull down the entire complexes. After extensive washing, the co-IP complexes were loaded on the gel and analyzed by immunoblotting. As shown in Figure 2, HuD was not identified in the co-IP complexes with the zipcode RNA while the tau RNA interacted concurrently with both HuD and ZBP1 proteins, in agreement with previous findings (32). These results suggested that ZBP1 and HuD binding to the zipcode is found to be competitive and mutually exclusive.

### Sequence and structural analysis of the *cis* mRNA localization signal of β-actin mRNAs

Extensive studies have shown that the zipcode element within the 3′ UTR of β-actin mRNA allows a specific binding of ZBP1 for its subcellular localization and translation control. A close examination of the zipcode sequence alignment of the β-actin 3′ UTRs among mammalian species showed an interspecies identity of 70% (Supplementary Figure S2). In particular, we found that a 5′-ACACCC-3′ sequence (shaded in red) and U-rich sequence UU(uu)UCU(u)U (shaded in blue), juxtaposed to 3′ end of the ACACCC within the zipcode, were highly conserved for all mammals examined, suggesting that these regions within the zipcode are functionally important. The ACACCC motif was originally discovered as a minimal *cis* localization sequence for β-actin mRNA in chicken fibroblasts *via* binding of ZBP1 protein (4,34–35). The ACACCC motif is totally conserved in all vertebrates aligned including birds, but both chicken and wild duck zipcode elements contain two copies of the ACACCC motif in tandem (shaded in yellow), in which the second one corresponds to the mammalian interspecies-conserved ACACCC motif. Since HuD has previously been reported to preferentially bind to U- or CU-rich sequences in the 3′ UTR of its target mRNAs in addition to ARE motifs, the U-rich sequence immediately distal to the ACACCC motif within the zipcode in mammals could serve as a binding site for HuD (19,32,36–38). However, other animals in *Chordata* including birds, reptiles, amphibians, and fishes examined do not seem to contain the U-rich or pyrimidine-rich sequences in the corresponding region of the mammalian β-actin 3′ UTR.

**Figure 3.**
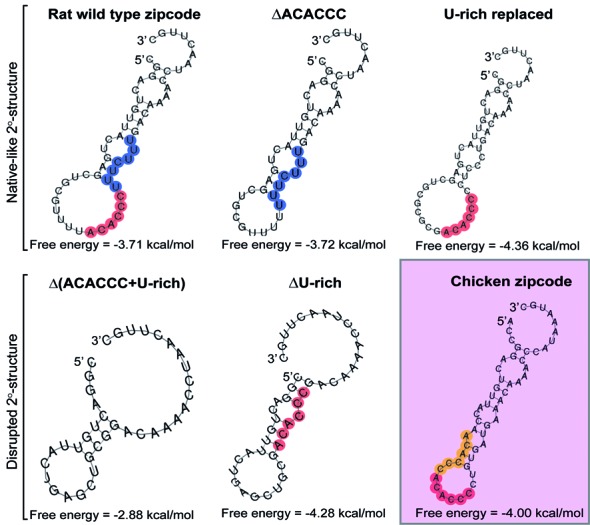
Secondary structures of the predicted zipcode RNAs. Secondary structures of representative examples of the wild type and mutated RNAs of the rat zipcode element were predicted by the RNA folding prediction software program Mfold. A secondary structure of the chicken zipcode RNA was also included (shaded in red). A species-conserved ACACCC motif was marked with red circles (and yellow in the chicken) and U-rich motif was marked on the figure with blue circles.

To search for putative secondary structure(s), the zipcode elements of rat and chicken β-actin 3′ UTRs were further analyzed with several web-server-based, RNA folding prediction algorithms such as Mfold and CentroidFold (Figure 3). A most common secondary structure of RNAs found in RNA folding prediction is a stem-loop structure, consisting of double-stranded RNA stems, unpaired internal bulges within the stem, a terminal loop, and an exterior loop at the 3′ end of the sequence. The parameters in the structure, including the length of the stem, the size of the terminal and exterior loops, and the number and size of bulges, are key determinants for specific interaction with its partner RBP (39). The rat/mouse zipcode RNAs were predicted to form a well-defined stem-loop structure containing two internal bulges, a 16-nt terminal loop, and a 3′ end 9-nt external loop. A similar structure displaying comparable features could also be obtained in the sequences of the chicken zipcode (Figure 3, shaded in red). The predicted secondary structure also showed that the ACACCC motif resides in the terminal loop region of the hairpin in the most thermodynamically stable structure. The folding free energy of the predicted secondary structures of the zipcode RNAs ranged from −3.71 kcal/mol to −4.36 kcal/mol, giving the highest concentration of the structured species in the test tubes with the assumption that the maximum number of base-pairing in the sequence gives the lowest free energy from the folded state. It was also noteworthy that the conserved U-rich sequence shaded in blue was predicted to form base pairs and a small bulge abutted of the predicted terminal loop.

### HuD protein binds a U-rich primary sequence, whereas ZBP1 recognizes secondary structure conformed by an RNA zipcode in the target transcript

**Figure 4.**
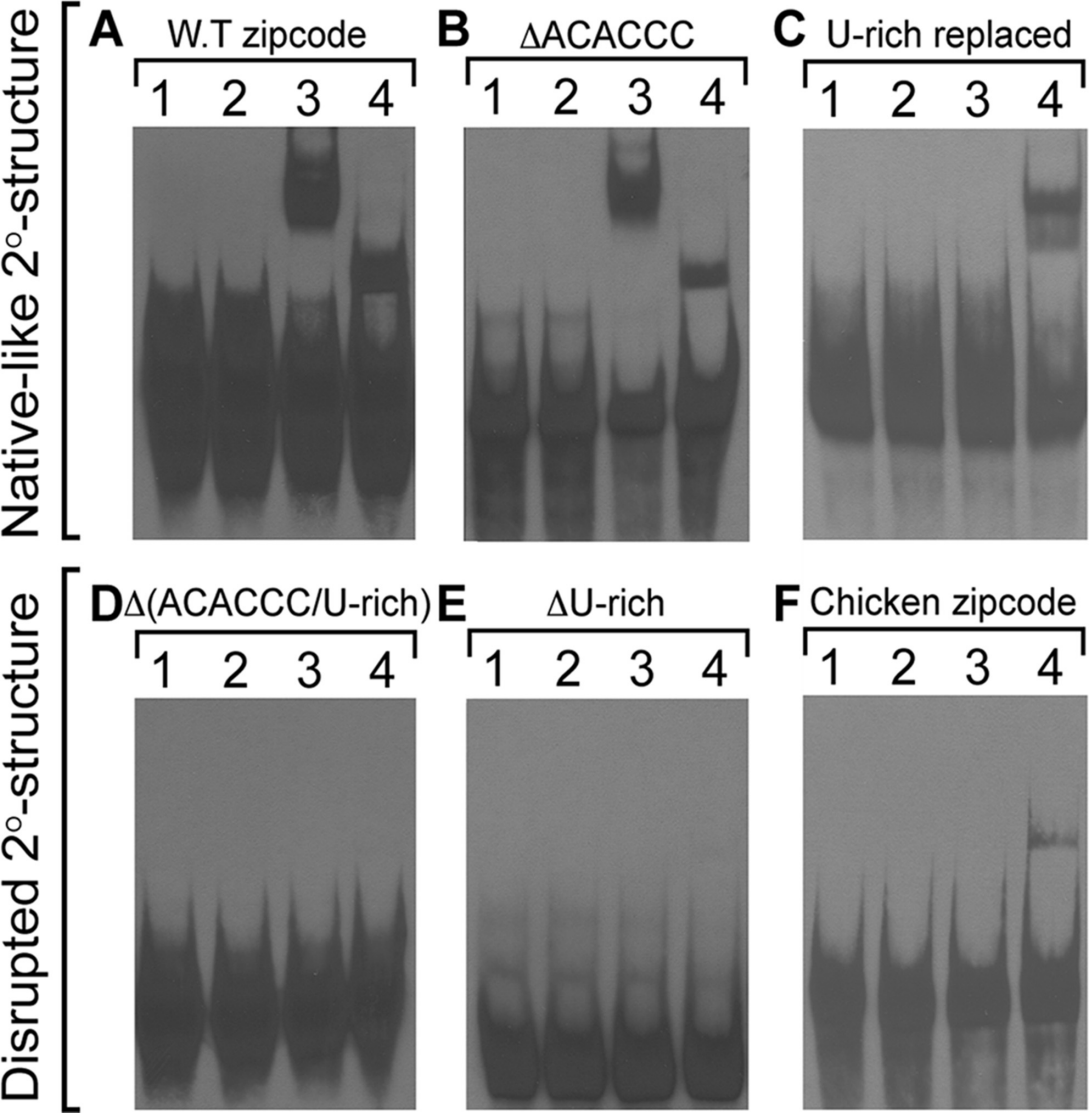
Representative REMSA gels of mutated zipcode RNAs with purified HuD and ZBP1 proteins. Binding of the wild-type zipcode RNA (**A**; WT zipcode) to HuD or ZBP1 was not significantly changed even when the interspecies-conserved ACACCC motif was deleted (**B**; ΔACACCC) but completely abolished when the U-rich motif was removed [**D**; Δ(ACACCC/U-rich) and **E**; ΔU-rich]. When the U-rich motif was carefully replaced to maintain the predicted secondary structure as that of the wild-type zipcode, HuD binding was completely inhibited with a reduced affinity to ZBP1 protein (**C**; U-rich replaced). Note that the purified rat ZBP1 protein recognized the chicken zipcode RNA, while purified rat HuD protein did not (**F**; chicken zipcode). Lane 1 contains only synthetic RNA, and lane 2 represents a control reaction that contained purified GST peptide with RNA; lane 3, purified HuD with RNA; lane 4, purified ZBP1 with RNA.

To investigate the recognition and binding preferences of the zipcode RNA by the two different RNA-binding proteins, HuD and ZBP1, we tested a series of sequence-mutated zipcode RNAs on their association with the purified HuD and ZBP1 proteins. First, in an effort to determine whether the primary sequence is sufficient for both HuD and ZBP1 binding, the conserved ACACCC or UUUCUUU motif of the rat zipcode element was simply deleted from the zipcode sequence, and REMSA experiments were carried out with purified proteins. Given the importance of ACACCC motif for ZBP1 recognition and binding (34,35), we expected to observe no binding or reduced affinity of ZBP1 to the ACACCC-deleted RNA. However, much to our surprise, the binding of not only HuD but also ZBP1 was not compromised when the ACACCC motif was removed from the rat zipcode sequence as compared with the wild-type RNA, suggesting that the primary sequence of the ACACCC motif itself is not essential for ZBP1 binding (Figure 4A; WT zipcode versus Figure 4B; ΔACACCC). This result was consistent with previous studies of isolated ZBP1 KH34 domains having no affinities for the deleted zipcode RNA fragment that contained the ACACCC motif (34). In contrast, the deletion of a conserved U-rich sequence from the zipcode RNA completely abolished HuD and ZBP1 binding (Figure 4E, ΔU-rich), suggesting that the U-rich sequence within the zipcode element is necessary for binding of both HuD and ZBP1. However, it was still possible to speculate that a plausible change in the RNA structure originating from the deletion of the conserved motifs causes changes in binding patterns. Taken together, these observations could be explained either by the structural basis of recognition of the target transcript both by HuD and ZBP1 proteins or their different modes of RNA recognition.

To determine if the integrity of the predicted secondary structure of the zipcode element is functionally important for both HuD or ZBP1 binding, the ACACCC motif or U-rich motif-deletion mutants of the zipcode RNAs were further analyzed by using the RNA folding prediction Mfold program. As shown in Figure 3 (ΔACACCC), we found a comparable secondary structure of the ACACCC-deleted mutant RNA displaying similar features with that of the wild type zipcode RNA. It seemed that the deletion of the ACACCC motif led to the predicted structure of the wild type zipcode except a smaller size of the terminal loop (a 10 nt-loop compared with a 16-nt loop). In contrast, the U-rich motif deleted-mutant (ΔU-rich) caused a noticeable change in the secondary structure with different features, including a decrease in the number of internal bulges, the size of the stem and the terminal loop, and an increased size of the 3′ end exterior loop, as compared with those of the wild-type RNA. These bioinformatics analyses suggested that an alteration to the secondary structure of the zipcode RNA resulting from the deletion of the U-rich motif could cause a clear change in the capability of both ZBP1 and HuD recognition and binding toward the zipcode RNA. The role of the secondary structure of the zipcode for ZBP1 and HuD binding was further examined by comparing the secondary structure of the double mutated version of this RNA by a simultaneous deletion of both the ACACCC and U-rich motifs [(Figure 3, Δ(ACACCC/U-rich)]. As predicted structure changed, the double-deletion mutant RNA did not form RNA-protein complexes with either protein (Figure 4D).

All of the above data indicated that the specific binding determinant(s) on the zipcode RNA for ZBP1 and HuD proteins seemed to be specific feature(s) with the secondary structure. To ascertain that the proposed secondary structure of the zipcode RNA displaying similar features with that of the wild type zipcode is sufficient for ZBP1 and HuD binding for subcellular localization, a mutant zipcode RNA was carefully designed, in which the conserved U-rich 5′-UUUCUUU-3′ sequence was replaced with 5′-CCUCCCU-3′, but the predicted structure was not disturbed (Figure 3, U-rich replaced). We also took consideration that the U-rich sequence, not a pyrimidine-rich sequence, is important for HuD binding. The secondary structure of this mutant RNA was predicted to display very similar features as in the wild type zipcode RNA. However, the U-rich replaced but structurally intact mutant zipcode RNA fully abolished the ability to bind to HuD, whereas ZBP1 binding was not significantly affected (Figure 4C). These results argued that only ZBP1 protein, but not HuD, requires the specific features within the secondary structure for recognition of the RNA. It also appeared that the primary sequence of the U-rich motif within the zipcode element provides the specificity of HuD for binding to the RNA.

**Figure 5.**
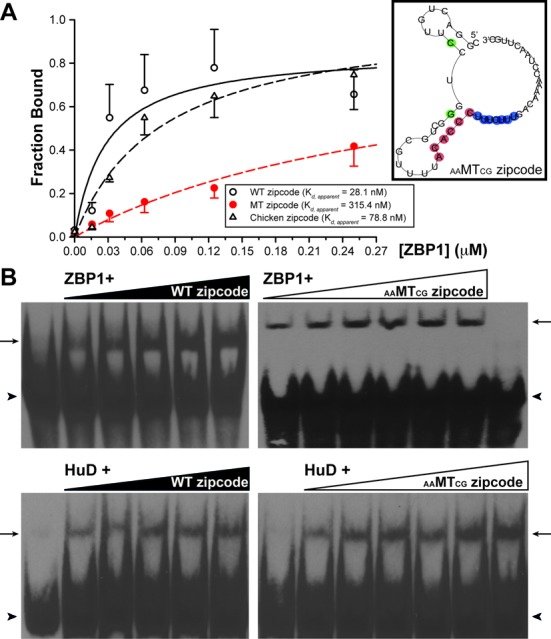
Different recognition motifs of ZBP1 and HuD for the same RNA. (**A**) Quantification of the fraction of zipcode RNAs bound in REMSA experiment. The measured values were fitted to a single site binding model using a nonlinear regression to determine a value for the apparent dissociation constant (*K*_d, apparent_) of rat ZBP1 binding to the wild-type rat zipcode (open circle), mutant zipcode (red circle), and wild-type chicken zipcode (triangle) RNAs. Error bars represented standard deviation (*n* = 3). The curves for the wild-type rat zipcode, mutant zipcode, and wild-type chicken zipcode RNAs corresponded to *K*_d, apparent_ = 28.1 nM, *K*_d, apparent_ = 315.4 nM and *K*_d, apparent_ = 78.8 nM, respectively. Inset shows a secondary structure of mutant RNA (_AA_MT_CG_ zipcode) predicted different from the wild-type RNAs. (**B**) Interaction between purified ZBP1 or HuD protein and the RNA was assayed in the presence of increasing amounts of the wild-type zipcode (WT zipcode; left panels) or the mutant zipcode RNA (_AA_MT_CG_ zipcode; right panels). Note that ZBP1 showed reduced affinity to the _AA_MT_CG_ zipcode RNA, but HuD showed no difference between the wild-type and mutant zipcodes. The triangles on the top of the gel images represented a serial dilution of the RNAs. Biotin-labelled free RNA (arrowhead) and RNA-Protein complexes (arrow) were indicated.

To further support the view that ZBP1 and HuD proteins recognize and bind the same target transcript in a fundamentally different manner with differential preference, we generated another form of mutant zipcode RNA (_AA_MT_CG_ zipcode), in which only the structure was potentially disrupted without changing the ACACCC and U-rich motifs (Figure 5A, inset). If ZBP1 in fact recognizes and binds the secondary structure of the target zipcode RNA, and HuD binding only requires the U-rich primary sequence in the zipcode, one would expect that the _AA_MT_CG_ zipcode RNA, in which the secondary structure is significantly disrupted but the U-rich sequence is kept intact, would show no affinity or at least reduced affinity to ZBP1 protein but would make no difference with regard to HuD binding. Figure 5A and B showed that ZBP1 displayed a 11-fold reduced affinity for binding to the structurally disrupted mutant zipcode RNA, as compared with that of the wild-type zipcode RNA (*K*_d_, _apparent_ = 315.4 nM for the mutant zipcode RNA versus *K*_d_, _apparent_ = 28.1 nM for the wild-type zipcode RNA). In contrast, purified HuD protein showed no apparent changes in the shifted gel mobility of the complex with the mutant RNA, compared to that with the wild-type zipcode (Figure 5B).

To test whether these predicted structures could be used as a reliable basis for understanding the structural aspects of RNA–protein interactions, the enzymatic probing analysis of RNA structure was carried out on the wild-type and mutant zipcode RNAs to experimentally verify the predicted structures (40,41). Since the formation and maintenance of the RNA structural integrity is determined primarily by base-paired elements, we hypothesized that the structural integrity could be evaluated by assessing the distinct electrophoretic separation footprint caused by differences in the cleavage products of specific RNases. Considering a rapid and straightforward analysis for determining which mutant zipcode RNAs are structurally distorted as compared to that of the wild-type zipcode, the RNAs were enzymatically digested in the presence of the double-strand-specific RNase III (Supplementary Figure S4). We observed that the mutant zipcode RNAs predicted to have disrupted secondary structures showed noticeable changes in the cleavage product patterns (indicated by asterisks in Supplementary Figure S4; lower panels), compared with those in the RNAs predicted to have a native-like secondary structure (Supplementary Figure S4; upper panels). Enzymatic probing of the RNA structures obtained with RNase III indicated pronounced changes that are consistent with predicted secondary structures.

Taken together these data indicate that while both ZBP1 and HuD proteins bind a *cis*-element zipcode in the 3′ UTR of β-actin mRNA, ZBP1 shows a recognition preference toward the structural basis, but that of HuD being toward a specific sequence preference, particularly, a U-rich sequence.

### HuD significantly influences stability of β-actin mRNA

**Figure 6.**
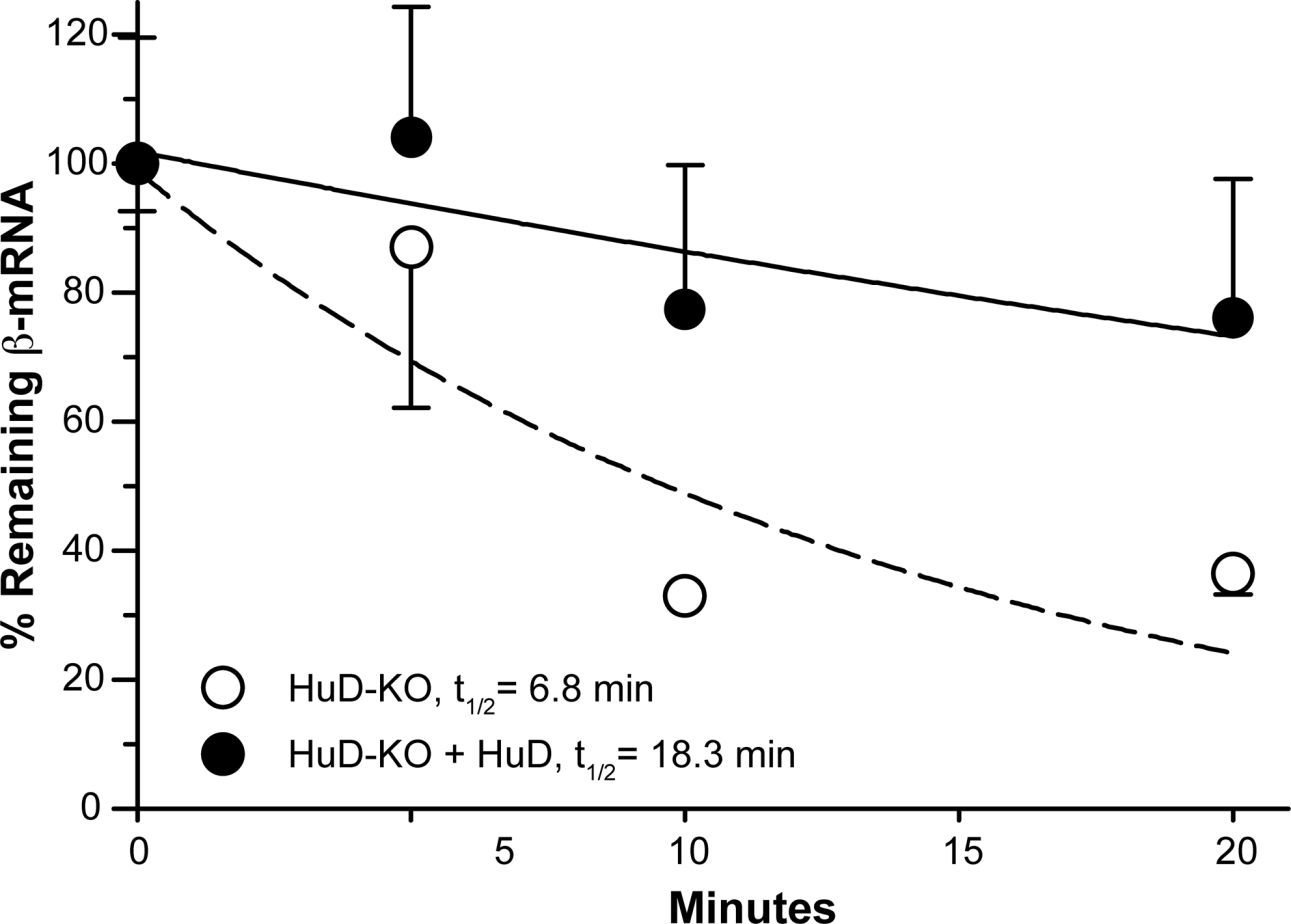
Influence of HuD on β-actin mRNA stability. Relative stability of β-actin mRNA was measured using S100 extracts from cortical tissues of HuD knockout (KD) mice. β-actin mRNA signals were normalized to GAPDH mRNA using the 2^−_(ΔΔ*C*t)_^ method and expressed relative to time = 0 min.

The Hu family is known to play roles in promoting mRNA stability (42–44). Our previous study showed that increased levels of HuD protein in the sciatic nerve following injury coincide well with the stabilization of GAP-43 mRNA (21). Both HuD and GAP-43 have been functionally implicated in neuronal differentiation, regeneration, and synaptic plasticity. However, HuD-dependent stabilization of β-actin mRNA has not been investigated per se. Therefore, we examined if HuD has an effect on β-actin mRNA stability using an *in vitro* RNA decay assay (Figure 6). Since S100 extract system has been shown to faithfully reproduce *in vivo* aspects of mRNA degradation (45,46), we utilized S100 extracts from *HuD^−/−^* KO mice. When S100 extracts were incubated without replenishing the HuD protein, β-actin mRNA decayed with a half-life of 6.8, which is relatively comparable to a time constant of 7.3 min (estimated to be a half-life of ∼5.5 min) for ‘unmasked’ β-actin mRNA reported in dendrites of cultured hippocampal neurons (47). In contrast, addition of purified HuD protein into the S100 extract showed β-actin mRNA half-life of 18.3 min, indicating that HuD stabilizes β-actin mRNA.

### Secondary structure of the zipcode of β-actin mRNA is necessary for axonal mRNA localization in DRG neurons

**Figure 7.**
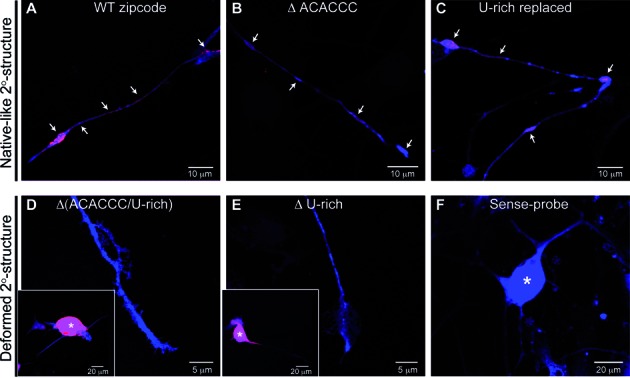
Validation of structural requirement of the zipcode RNA for axonal localization. Panels (**A**)–(**C**) showed representative images of DRG neurons expressing GFP^myr^zipcode^wild-type^, GFP^myr^zipcode^ΔACACCC^, and GFP^myr^zipcode^U-rich replaced^ constructs, respectively. GFP mRNA (red) and protein (blue) signals were observed in both the axons and growth cones (arrows) of DRG neurons transfected with reporter constructs containing the native-like secondary structure. However, those with the disrupted secondary structure [**D**; GFP^myr^zipcode^Δ(ACACCC/U-rich)^, **E**; GFP^myr^zipcode^ΔU-rich^] showed reporter mRNA in the cell body only (asterisks represented cell body in insets). Sense cRNA riboprobe for GFP showed no signal in exposure-matched images of GFP^myr^zipcode^wild-type^ transfected DRGs (**F**).

The zipcode in the 3′ UTR of β-actin mRNA has been shown to be both necessary and sufficient for localization of the mRNA into leading edge of fibroblasts and distal axons of neurons (4–5,35). To address the physiological relevance of the zipcode element for the axonal localization of the transcript and whether the mutated zipcode without the structural disruption is still capable of localizing the mRNA into distal axons of dorsal root ganglion (DRG) neurons in culture, we generated myristoylated GFP reporter constructs carrying the mutated zipcodes we tested above. Adult rat DRG neurons were transfected with each of these constructs, and fluorescence *in situ* hybridization (FISH) was used to directly visualize reporter mRNAs with cRNA probe specific for the reporter mRNA. We first checked to see if all of these reporter mRNAs are expressed in the transfected DRG neurons by focusing on GFP mRNA FISH signals in cell bodies (Figure 7D and E, insets). We were able to detect strong mRNA signals for all of these constructs tested in cell bodies. Although the intensity of mRNA signals in distal processes and axons was relatively weaker compared with that in cell bodies, GFP mRNA was detected in axons of neurons transfected with the construct carrying the wild-type zipcode and the mutated zipcodes that maintain the native-like secondary structure (Figure 7A, B, and C). These data are consistent with the ZBP1 binding to the 3′ UTR of β-actin mRNA for transport into axons through its zipcode element. Therefore, we next asked if ZBP1 binding to RNA alone is sufficient for its localization. Given the specific binding preference of ZBP1 for the secondary structure of the RNA, we generated a GFP reporter construct, in which the HuD binding was inhibited by substituting the U-rich motif, but ZBP1 binding was not affected by maintaining the secondary structure (Figures 3 and 7C, U-rich replaced). As shown in Figure 7C, DRG neurons expressing the native-like secondary structure of the zipcode without having a U-rich sequence for HuD binding showed no noticeable difference in axonal FISH signal, compared with the wild-type zipcode expressing neurons. However, neurons expressing mutant zipcodes with disrupted secondary structure showed no axonal GFP mRNA signal, whose subcellular distribution has been shown to be restricted in cell bodies (Figure 7D and E). These data suggested that ZBP1 binding to RNA is necessary and sufficient for localization into axons.

Although the data above directly link ZBP1 to mRNA localization into axons, we previously showed that axonal localization of GAP-43 mRNA requires an ARE element, presumably for HuD binding. Therefore, we tested if HuD alone might similarly be sufficient for localizing GFP mRNA into axons. For a direct test for the ability of HuD binding alone to localize reporter mRNA into axons, we generated a GFP reporter with the zipcode where HuD binding site, a U-rich sequence, was intact but the secondary structure for ZBP1 binding was disrupted (Supplementary Figure S5). DRG neurons transfected with this construct showed only weak axonal mRNA signals compared with neurons expressing wild-type zipcode, indicating that HuD binding alone is not efficient for targeting reporter mRNA into axons. These observations were also consistent with the reduced K*_d_* of the structurally disrupted RNA for ZBP1 binding (Figure 5).

## DISCUSSION

We present here that HuD primarily recognizes and binds to the primary sequence feature in the zipcode of rat β-actin mRNA, whereas the ZBP1 preferentially requires a proper spatial arrangement of the zipcode for binding, as previously determined (48,49). Furthermore, we demonstrated that the HuD recognizes and binds the U-rich sequence following the ACACCC motif within the zipcode, which is presumably overlapped with ZBP2/KSRP recognition site. Nucleotide substitution of the zipcode sequence or deletion of the core ACACCC motif does not seem to affect the binding capability of the ZBP1 as long as the RNA maintains the predicted proper structure. When the predicted structure of the zipcode was significantly disrupted by nucleotide substitution or deletion, the ZBP1 no longer bound or significantly reduced the affinity to the RNA. These results are strongly consistent with the previous structural analyses of ZBP1 protein regarding the interaction with the chicken zipcode RNA (34,49). In contrast, changes in the U-rich sequence without affecting the structure dramatically reduce only HuD binding for the RNA (Figure 4C). We propose that the zipcode core ACACCC primary sequence *per se* in the rat β-actin mRNA's 3′ UTR is not sufficient for binding of ZBP1 to target its RNA to the final destination, and that ZBP1 and HuD recognize the same target zipcode RNA in a fundamentally different way with differential relative preference.

### HuD binds to U-rich sequence in the 3′ UTR of rat β-actin mRNA

HuD binding to ARE-containing mRNAs leads to stabilization of the transcripts. Of HuD target transcripts, candidate plasticity-related gene 15 (cpg15, also known as neuritin) mRNA interacts with HuD *via* AREs in the 3′ UTR and forms an mRNP complex with the survival of motor neuron (SMN) protein. HuD-SMN complex then transports the associated cpg15 mRNA to axonal processes of spinal motor neuron, as well as regulates the local stability or translation, or both. SMN can also bind hnRNP-Q/R protein, which localizes β-actin mRNA to axonal growth cones of motoneurons (3,50–52). Although we have previously demonstrated that β-actin mRNA is present in HuD immunoprecipitate, there has been no direct evidence of how HuD recognizes and binds to β-actin mRNA. In the present study, using purified HuD protein and synthetic zipcode RNA, we demonstrated that HuD recognizes and directly binds to the zipcode of rat β-actin mRNA's 3′ UTR. Given that ARE regulatory sequences consist of uridine- or adenine/uridine-rich stretches, the initial simple mutation of uridines in the U-rich sequence following the ACACCC motif without affecting the predicted structure completely abrogated the binding of HuD to the RNA, while ZBP1 binding was not affected, suggesting that HuD recognizes and binds preferentially a U-rich motif in the target zipcode RNA. This is consistent with a previous report, in which HuD binds U-rich target mRNA with higher affinity than AU-rich sequences (53).

ZBP2, a chicken homologue of human KH-type splicing regulatory protein (KSRP), is known to participate in destabilizing its target mRNAs (22,54–55). Singer and colleagues reported that ZBP2 plays a role in transporting β-actin mRNAs to neuronal processes by binding to the second ACACCC motif of the two copies in the chicken zipcode or presumably to a pyrimidine-rich sequence following the ACACCC motif in mammalian zipcode, in which only one copy of ACACCC is present (34–35,48–49). However, they observed no simultaneous association of ZBP1 and ZBP2 with the zipcode RNA and suggested a ‘handover’ model in which ZBP2 and ZBP1 bind sequentially to closely apposed but distinct binding sites. That is, ZBP2 cotranscriptionally recognizes and binds nascent β-actin mRNA first and then passes the bound transcript on to ZBP1 later for exporting to cytoplasm and subsequently for transporting to distal processes of neurons (48). In this ‘handover’ model, the binding of ZBP2 to the zipcode seems to be prerequisite for the efficient binding of ZBP1 later. However, the competitive binding of multiple RBPs would be also expected, especially considering that the binding sites for these RBPs are overlapped or closely apposed to one another. If ZBP2 binding to the zipcode is indeed a prerequisite for the ZBP1 binding to the core motif, and the binding site is completely overlapped or shared with HuD, then HuD could regulate ZBP1 binding and subsequent transport of β-actin mRNA to processes of neurons at multiple steps. For example, HuD could initially play a role in the nucleus by a competition against binding of ZBP2 for the β-actin mRNA cotranscriptionally (Supplementary Figure S6). Alternatively, HuD could competitively bind the pyrimidine (U) rich motif of the zipcode and directly inhibit binding of ZBP1 to β-actin mRNA in the cytosol, eventually decreasing levels of β-actin mRNA in axons. It is also plausible that the binding of HuD to the zipcode in the cytoplasm might be involved in mRNP remodelling that regulates the recruitment of molecular motors for axonal localization. It is noteworthy that ZBP1 binding to the zipcode RNA *in vitro* was not affected by the absence of ZBP2 but drastically hampered by HuD (Figure 1B), indicating that a direct competition between ZBP1 and HuD for the overlapped binding site within the zipcode is conceivable. Surprisingly, purified rat HuD protein did not show an affinity to chicken zipcode RNA (Figure 4F), implicating that HuD preferentially recognizes Us rather than Cs in the pyrimidine-rich motif. The evolutionary pressure to change these sequence differences may confer unique regulatory properties of HuD in different steps of RNA metabolism including stability, localization, and translation in mammals (Supplementary Figure S7). How these distinct properties of HuD protein translate into biological specificity within the neuron, particularly regarding axonal localization of mRNAs, however, still remains to be examined.

The majority of ARE-binding proteins including AU-binding factor 1 (AUF1; also known as hnRNP D), KSRP, tristetraprolin (TTP), T-cell-restricted intracellular antigen 1 (TIA-1), and TIA-1-related protein (TIAR) has been implicated in mRNA degradation and/or translational repression. However, the ELAV/Hu proteins positively regulate the stability and translation of their target mRNAs (56–58). Consistent with these studies, our data in the present study showed that HuD positively influences β-actin mRNA stability (Figure 6). However, HuD-dependent regulation of β-actin mRNA stability may not be the only molecular mechanism modulating expression of ARE-mediated genes. As RBPs such as ZBP1 and ZBP2 directly target β-actin mRNA into its final subcellular destination by binding to the zipcode *cis*-element within the 3′ UTR (4,35,59–60), the HuD binding site adjacent to the zipcode raised the possibility that HuD could regulate the distribution of β-actin mRNA in collaboration with ZBP1 and/or ZBP2 proteins. Although our data showed that ZBP1 and HuD binding to the zipcode RNA is mutually exclusive in cell-free systems, as shown in previous studies (48,61), we are uncertain to what extent these *in vitro* findings were applicable *in vivo*. For example, in intact cells HuR and AUF-1 concurrently bind on the same target transcript, rather than competing for ARE binding *in vitro* (62,63). Therefore, the molecular mechanism of this effect is still plausible. Given that Hu proteins move between different RNP complexes including stress granules and actively shuttle between nucleus and cytoplasm under certain conditions (64,65), it is possible that the reversible association between HuD and the β-actin mRNA *in vivo* helps precisely control local levels of the mRNA by regulating mRNA turnover and/or localization in appropriate subcellular regions depending on cellular conditions. Although HuD has been positively associated with mRNA stability, it is not clear whether HuD remains bound to β-actin mRNA during transport. Thus, testing this molecular mechanism that HuD is an active transport factor will help to elucidate how the neuron integrates RBP-mediated posttranscriptional regulator and control of local gene expression in response to local signals.

### HuD and ZBP1 bind the zipcode RNA target in a fundamentally different way

Spatial and temporal restriction of gene expression to specific subcellular domains, especially of neurons, is critical to achieve autonomous response to external stimuli. Increasing evidence demonstrates that specific interactions between RNA localization elements and cellular factors play an essential role in cytoplasmic sorting of mRNAs to their destination (66–68). These RNA localization elements seem much more heterogeneous in size and structure than previously thought. They can be ranged from several nucleotides in length up to very complex structural levels. For example, mRNA encoding myelin basic protein in oligodendrocytes contains two partially overlapping 11-nt *cis*-element in its 3′ UTR (69). We previously showed that 40-nt ARE within the 3′ UTR of GAP-43 mRNA is sufficient for axonal localization in adult sensory neurons (21). It is also likely that localized transcripts contain more than one copy of localization *cis*-element as well as a combination of different zipcodes mediating distinct steps in localization (70). Complexity can be further increased if these signals (partially) overlap and are recognized by different RBPs. Because of the complexity and the variability of the localization sequences identified to date, it has not yet been possible to unambiguously identify localization elements by computational prediction or even to deduce common sequence and structural motifs.

In addition to the localization sequence elements of mRNAs, the stability, transport, and translation of an mRNA are determined, to a large extent, by dynamically interacting *trans*-acting factors including not only RBPs but also small non-coding regulatory miRNAs (43–44,71–73). The molecular mechanisms involving direct binding of the mRNA by *trans*-acting factors are particularly important for highly polarized neurons, in which specific neuronal mRNAs have to be stabilized to survive and translationally repressed during their transport. To this end, the interactions of mRNAs with RBPs and/or miRNAs involve the functional cooperation between different *trans*-factors that can work in collaboration or exert opposing effects on target mRNAs. As detailed in Supplementary Figure S6, ZBP1 and HuD might compete for binding to a single *cis*-element but co-reside on the same mRNA as it is transported from its site of synthesis in the nucleus to its final destination.

To obtain further insight into the interaction modes of HuD and ZBP1 with the same zipcode RNA *in vitro*, we employed RNA electrophoretic mobility shift assay with purified proteins and multiple synthetic RNA substrates whose structures were predicted with RNA folding prediction algorithms. Although our understanding of how RBPs recognize/bind their target RNAs is still very limited and the capability to predict which RBP binds which RNA is not in any way plausible, relatively recent biochemical studies by Singer and coworkers showed that the spatial orientation of ZBP1 KH domains 3 and 4 at opposing ends of ZBP1 leads the chicken zipcode RNA to loop around ZBP1 to an anti-parallel orientation (34,49). While some RBPs such as ZBP1 recognize their binding sites within a secondary structural level, others such as members of the PUF protein family can bind specific unstructured sequence in RNA (74–76). In the latter RBPs, recent algorithms developed to identify secondary structures that can be recognized by RBPs are not likely applicable for the RBPs (77,78). In contrast to ZBP1, we found that HuD exhibited the recognition preference for a U-rich sequence following the zipcode core ACACCC motif, especially considering that several studies using (RNA) immunoprecipitation and pull down assays have shown that ZBP1 and HuD are mutually exclusive of each other in the mRNP composition (20,79). Thus, overall, these results strongly support the view that the RNA-binding proteins ZBP1 and HuD interact with RNA targets in a fundamentally different manner.

Taken together, these results provide an experimental basis suggesting that the interplay of multiple RBPs for the target RNAs can play unique roles, independently or cooperatively, in mRNA metabolism including stability, localization, and translation, as well as allow effective redistribution of the mRNAs in response to local stimuli. Of course, understanding their interaction relating to mRNA metabolism for normal biological properties and, most importantly, for an eventual disease context, will require future analyses.

## SUPPLEMENTARY DATA

Supplementary Data are available at NAR Online.

SUPPLEMENTARY DATA
