# Supplementary material for: Different motif requirements for the localization zipcode element of β-actin mRNA binding by HuD and ZBP1
Source: Nucleic Acids Res. 2015 Jul 7;43(15):7432–46. doi: 10.1093/nar/gkv699 (PMC4551932; doi:10.1093/nar/gkv699)
Supplement: SUPPLEMENTARY DATA [file supp_43_15_7432__index.html]

Different motif requirements for the localization zipcode element of β-actin mRNA binding by HuD and ZBP1 — Different motif requirements for the localization zipcode element of β-actin mRNA binding by HuD and ZBP1 — SUPPLEMENTARY DATA 

# Different motif requirements for the localization zipcode element of β-actin mRNA binding by HuD and ZBP1

## SUPPLEMENTARY DATA

- SUPPLEMENTARY DATA
