# Supplementary material for: Different motif requirements for the localization zipcode element of β-actin mRNA binding by HuD and ZBP1
Source: Nucleic Acids Res. 2015 Jul 7;43(15):7432–46. doi: 10.1093/nar/gkv699 (PMC4551932; doi:10.1093/nar/gkv699)
Supplement: SUPPLEMENTARY DATA [file supp_gkv699_nar-00503-r-2015-File009.pdf]

Supplementary Data

Figure S1

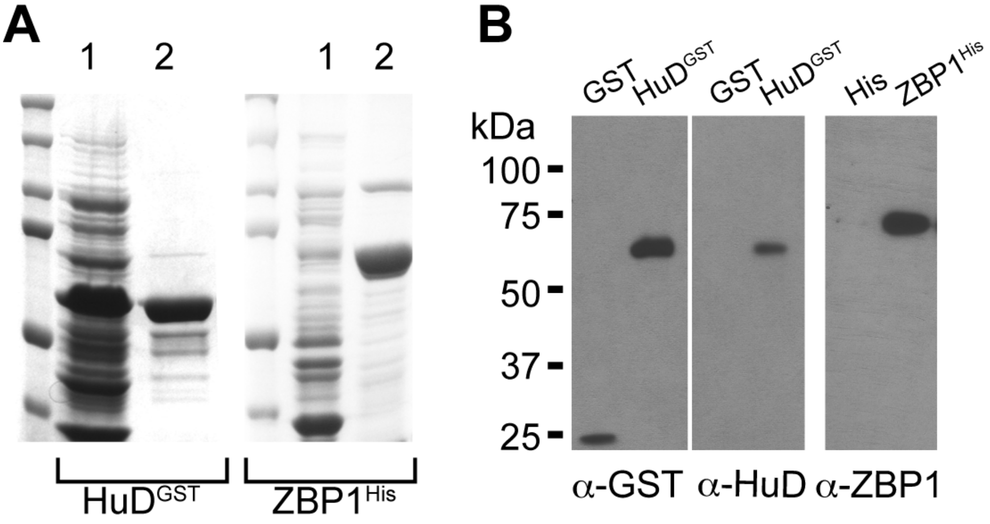

Figure S1. Purification of HuD and ZBP1 proteins. GST-tagged HuD (HuD^{GST}) and His-tagged ZBP1 (ZBP1^{His}) proteins were expressed in BL15 *E. coli* expression strain and purified using Glutathione-Sepharose beads and Ni-NTA beads, respectively. (**A**) Crude protein extracts (lane 1) and purified proteins (lane 2) were electrophoresed onto 10% SDS-PAGE and visualized by Coomassie blue staining. (**B**) Western blotting analysis was performed using anti-GST (α-GST), -HuD (α-HuD), and -ZBP1 (α-ZBP1) antibodies. Purified controls (GST and His), a GST-tagged HuD (HuD^{GST}), and a His-tagged ZBP (ZBP1^{His}) proteins were loaded on gels and blotted onto PVDF membranes. Left panel, the blot with purified control GST and HuD^{GST} proteins was initially probed with α-GST antibody. Middle panel, the same blot was subsequently stripped and reprobed with α-HuD antibody to detect tagged protein. Right panel, the blot with purified control His and ZBP1^{His} was probed with α-ZBP1 antibody. Standard molecular markers are indicated on the left.

Figure S2

```
T-COFFEE multiple sequence alignment, Version_10.00. r1613
Cedric Notredame

Human           GG--CGGACUAUgACU---UAGUU--GCGU---UACACCCUU--UCU--UGACA---AAACCUA--ACU--UGC 53
Chimpanzee      GG--CGGACUGUgACU---UAGUU--GCGU---UACACCCUU--UCU--UGACA---AAACCUA--ACU--UGC 53
Cow             GG--CGGACUGUUAgc-------U--GCGU---UACACCCUUuuUCU--UGACA---AAACCUA--ACU--UGC 51
Sheep           GG--CGGACUGUUAgc-------U--GCGU---UACACCCUUuuUCU--UGACA---AAACCUA--ACU--UGC 51
Wolf            GG--CGGACUGUUACUu--UAGUU--GCGU---UACACCCUU--UCU--UGACA---AAACCUA--ACU--UGC 54
Rabbit          GG--CGGACUGUUAgag-ccAGUg--GCGg---gACACCCUC--UCU--CGACg---AAACCUA--AC---gGC 54
Treeshrew       GG--CGGACUGUUACU---UAGUU--GCGU---UACACCCUU--UCU--UGACA---AAACCUA--ACU--UGC 53
Guinea_pig      GG--CGGACUGUUACUacuUuGcU--GCGU---UACACCCUU--UCU--UGACAaaaAAACCUA--ACU--UGC 59
Rat             GG--CGGACUGUUACU---gAGcU--GCGUu-uUACACCCUU--UCU-uUGACA---AAACCUA--ACU--UGC 56
Mouse           GG--CGGACUGUUACU---gAGcU--GCGUu-uUACACCCUU--UCU-uUGACA---AAACCUA--ACU--UGC 56

Chicken         AC--CGGACUGUUACC---AACAC-----C---CACACCCcUg-UGaugAAACA---AAACCCA-uAAa--UGC 54
Wild_duck       AC--CGGACUGUUACC---AACAC-----C---CACACCCUUg-UGacaAAAug---AAACCCAuAAAUgcgug 57
Alligator       AC--CGGACUGUUACC---A-Cg------u---CACACCg----a-----AACA---AgAACCC-A------ua 40
Xenopus         AggaCaGACccUUuCa----ACAu--GaaCaaaUgUACC-Ug--U-g--cAggA---AgAUCaC--AuU--gGC 55
Trout           AC---aGACUGUacCC---AuCcCaaaCGa---CcgACCCagccaCacccG------AcuACCA-c-uU--cag 55
Zebrafish       Aa--CGGACUGUUACC---AcuuC--aCG----CcgACuCaaacUGcgcAGA-g---AAACuUC-a-Aa--cGa 55
                      ***  *                        **                   *
```

Figure S2. Alignment and comparison of the nucleotide sequence of the zipcode RNA. Multiple sequence alignment of the zipcode homologues across species with T-COFFEE showed a high degree of sequence similarity, suggesting an interspecies-conservation of the zipcode. The yellow and red boxes corresponded to the first and second copy of the ACACCC motif, respectively, present in the chicken zipcode, and the blue box represented a conserved U-rich sequence in mammals.

Figure S3

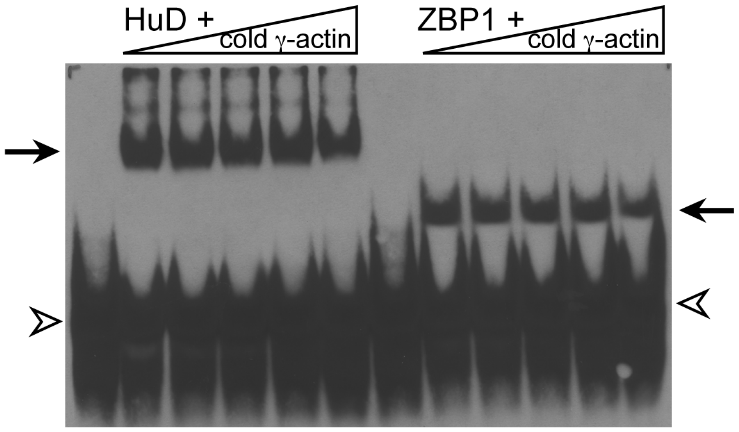

Figure S3. Representative RNA-EMSA gels with unlabeled nonspecific γ-actin RNA. The addition of an excess (increasing concentration up to >200-fold molar) of unlabeled nonspecific γ-actin 3'UTR RNA did not compete with specific interactions of the zipcode RNA for purified HuD- (left) or ZBP1-protein (right), and the shifted bands were preserved. The triangles on the top of the images represented a serial dilution of unlabelled γ-actin RNA. Open arrowheads indicated biotin-labelled free zipcode RNA and arrows indicated the RNA-protein complex.

Figure S4

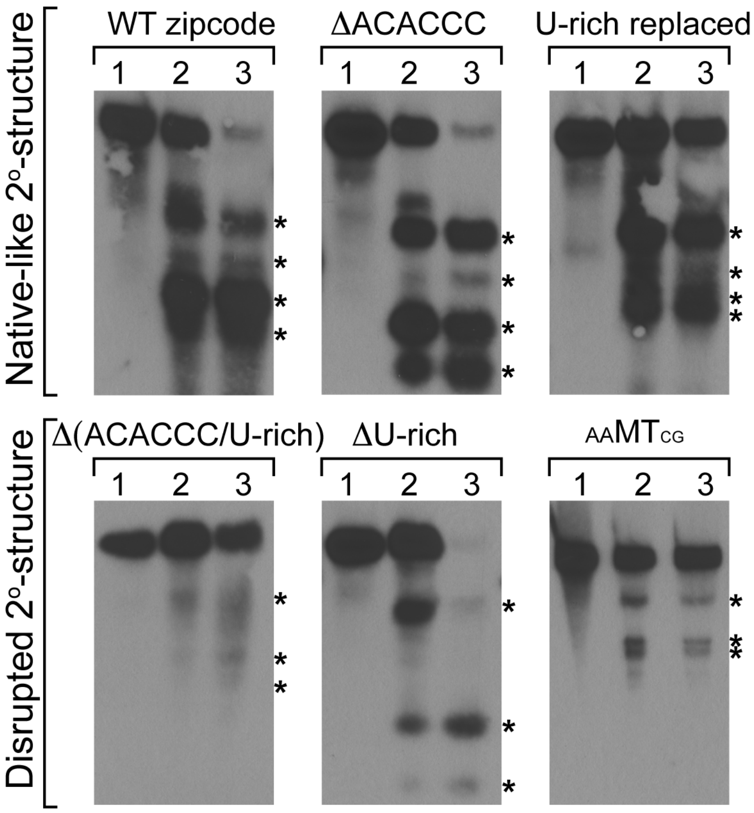

Figure S4. Enzymatic probing analysis of the zipcode RNA structures. Biotin-labelled zipcode RNAs were cleaved using 0.4 and 4 units of the double strand-specific RNase III (lanes 2 and 3, respectively) for 20 min at 37°C. Uncleaved RNA was shown in lane 1. Cleavage products of RNase III were indicated by the asterisk. Note that four bands were clearly visible from the RNAs predicted to have a native-like secondary structure, while only three bands were detected in the cleavage products from the mutant RNAs predicted to have a disrupted secondary structure.

Figure S5

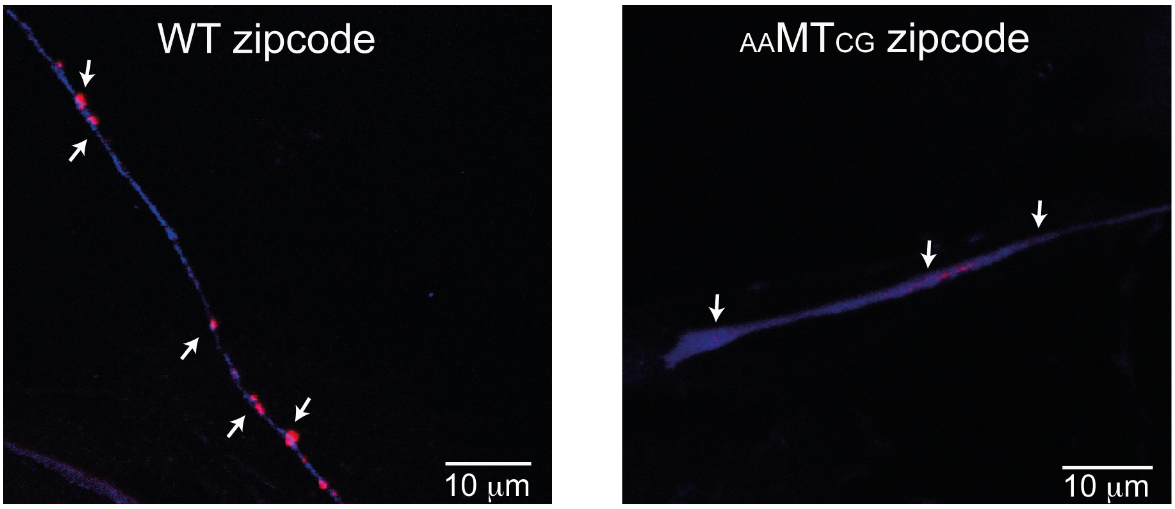

Figure S5. Representative exposure-matched images of GFP mRNA (red) and NF protein (blue) in axons of DRG neurons transfected with reporter constructs containing the wild type or mutant zipcode element. Neurons expressing the mutant zipcode RNA with a significantly disrupted secondary structure showed significantly weak reporter mRNA signals (arrows) in the axons, compared with those containing the wild-type zipcode.

Figure S6

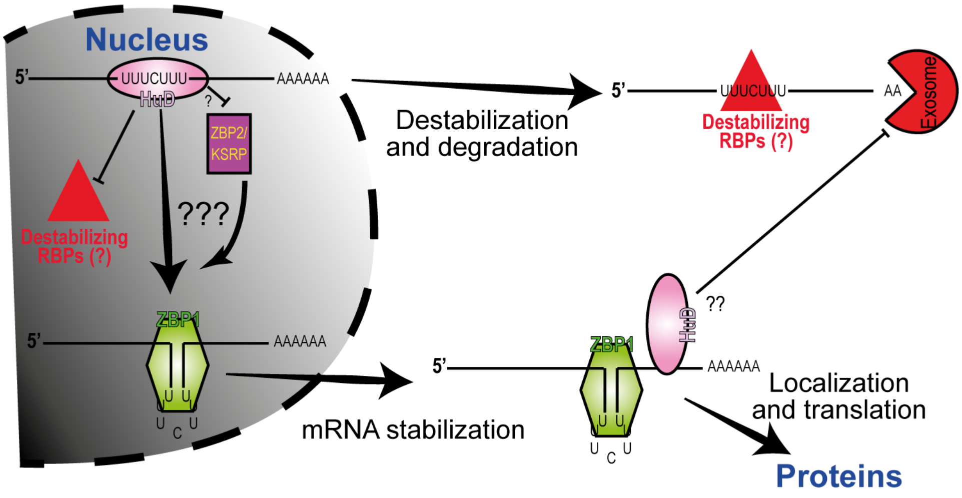

Figure S6. A model illustrating post-transcriptional regulation of localizing mRNAs in neurons. HuD binding to the *cis*-element stabilizes its target transcript by competing with destabilizing RBPs. The messenger ribonucleoprotein (mRNP) complex containing ZBP1 in the cytoplasm might also bind to HuD protein, and transport translationally silent mRNA into its final destination.

Figure S7

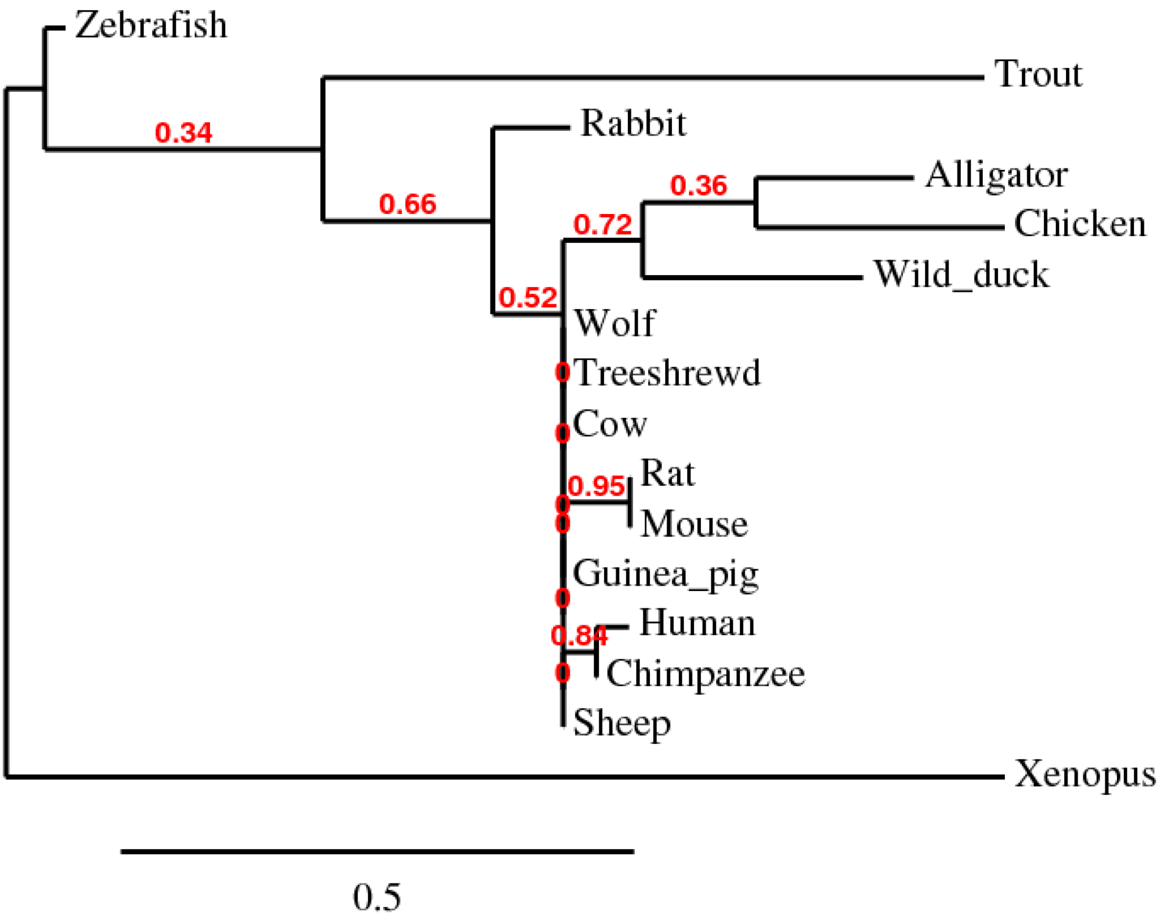

Figure S7. The phylogenetic tree of the β-actin mRNAs' zipcode. Multiple sequence alignment of the zipcode homologues across species was done using ClustalW. The phylogenetic tree of the zipcode was generated using *Phylogeny.fr* (www.phylogeny.fr) (80) based on the nucleotide sequence. The higher the value next to each node in red (between 0 and 1), the stronger the evidence that the sequences to the right of the node cluster together to the exclusion of any other. The scale bar at the bottom showed substitution rate of nucleotides per site required to generate the corresponding tree.

References

80. Dereeper, A., Guignon, V., Blanc, G., Audic, S., Buffet, S., Chevenet, F., Dufayard, J.F., Guindon, S., Lefort, V., Lescot, M. *et al.* (2008) Phylogeny.fr: robust phylogenetic analysis for the non-specialist. *Nucleic Acids Res*, **36**, W465-469.
